# Experiences of Clinical Clerkship Students With Mindfulness-Based Stress Reduction: A Qualitative Study on Long-Term Effects

**DOI:** 10.3389/fpsyg.2022.785090

**Published:** 2022-03-31

**Authors:** Inge van Dijk, Maria H. C. T. van Beek, Marieke Arts-de Jong, Peter L. B. J. Lucassen, Chris van Weel, Anne E. M. Speckens

**Affiliations:** ^1^Radboudumc Center of Mindfulness, Department of Psychiatry, Radboudumc, Nijmegen, Netherlands; ^2^Department of Psychiatry, Radboudumc, Nijmegen, Netherlands; ^3^Department of Primary and Community Care, Research Institute of Health Services, Radboudumc, Nijmegen, Netherlands; ^4^Department of Health Services Research and Policy, Australian National University, Canberra, ACT, Australia

**Keywords:** mindfulness, positive psychology, positive education, wellbeing, medical student, qualitative research

## Abstract

**Purpose:**

To explore the mindfulness practice, its long-term effects, facilitators and barriers, in clinical clerkship students 2 years after participation in an 8-week mindfulness-based stress reduction (MBSR) training.

**Method:**

A qualitative study was performed by semi-structured in-depth interviews with 16 clinical clerkship students selected by purposive sampling. Students had participated in a MBSR training 2 years before and were asked about their current mindfulness practice, and the long-term effects of the MBSR training. Thematic analysis was conducted using the constant comparison method. Data saturation was reached after 16 interviews.

**Results:**

Most interviewees were still engaged in regular, predominantly informal, mindfulness practice, although some discontinued mindfulness practice and reported an “*unchanged lifestyle*.” Three main themes came forward; (1) “*focused attention and open awareness*” during daily activities as core elements of long-term mindfulness practice; (2) “*changes in behavior and coping*” that resulted from taking a pause, reflecting, recognizing automatic behavioral patterns and making space for a conscious response; (3) “*integration in personal and professional life*” by enhanced enjoyment of daily activities, improved work-life-balance and making different career choices. Barriers and facilitators in starting and maintaining mindfulness practice were (1) understanding and intention as “*pre-conditions*”; (2) practical, personal, and professional factors of students in maintaining practice.

**Conclusion:**

Two years after participation in a MBSR training, many interviewees were still engaged in (mostly informal) mindfulness practice contributing to both personal and professional changes. In light of the high clerkship demands, MBSR training could be a valuable addition to medical curricula, supporting medical students in developing necessary competencies to become well-balanced professionals.

## Introduction

Managing the high demands of healthcare requires physicians to be well-balanced and healthy. The CANMEDS physician competency framework, which is currently used in many medical curricula worldwide, describes a “medical expert” as a communicator, collaborator, leader, health advocate, scholar, and professional (Frank et al., 2015).

Physicians’ commitment to their own positive (mental) health and wellbeing is part of the “professional role.” Especially, when considering that physicians (in training) and medical students are exposed to multiple stressors of academic, clinical, and psychosocial origin, including high workload, exposure to human suffering and death, and difficulties in work-life balance. High levels of stress in these professionals are associated with symptoms of depression and burn-out, and lower self-reported quality of life (Prins et al., 2010; Haldorsen et al., 2014; Naji et al., 2021), in particular millennials (Hill et al., 2018). Psychological distress in medical students and residents is associated with a decrease in empathy (Neumann et al., 2011), which may negatively affects quality of patient care and safety (Shanafelt et al., 2002). Importantly, training in (self-)compassion could promote medical students’ health and enhance clinical care (Weingartner et al., 2019; Dev et al., 2020). Although self-awareness is explicitly mentioned in the CANMEDS framework, most medical curricula do not provide (explicit) tools for developing this skill. A qualitative study in medical and surgical residents indicated that Mindfulness-Based Stress Reduction (MBSR) training could serve as a tool to cultivate professional competencies like (self-) awareness, self-acceptance, resilience, self-care, and work-life balance (Verweij et al., 2018).

Mindfulness-based stress reduction is a standardized 8-week group training developed by Jon Kabat-Zinn to improve self-awareness and compassion, including formal meditation, psycho-education, and practice integrated in daily activities (Kabat-Zinn, 1982). Participants are encouraged to change unhelpful automatic patterns, enhance self-care and adopt a non-judgmental attitude. A recent review investigating benefits of MBSR training for undergraduate medical students including nine studies showed that the training is associated with improved psychological wellbeing and self-compassion, although effects on empathy were mixed (Polle and Gair, 2021). A recent Cochrane review (Kunzler et al., 2020) on psychological interventions to promote resilience in healthcare students reported five published studies (Warnecke et al., 2011; Erogul et al., 2014; Smeets et al., 2014; Galante et al., 2018; Barry et al., 2019) focusing on mindfulness interventions. The results indicated positive effects on resilience, anxiety, and depression, but more research is needed particularly with a longer follow-up period.

Until now, most research about mindfulness interventions is performed in undergraduate students, while research in clinical clerkship students seems to be scarce. One study in clinical clerkship students reported that an adapted 4-week twice a week mindfulness elective resulted in a reduction of depression, emotional exhaustion and perceived stress as well as an increase of self-compassion and mindfulness (Garneau et al., 2013). A randomized controlled trial (RCT) demonstrated that clerkship students completing MBSR training reported a reduction of psychological distress and an improvement of positive mental health over the course of a 20-month follow-up period (van Dijk et al., 2017).

Moreover, quantitative research has dominated this field thus far (Dobkin and Hutchinson, 2013). Two qualitative studies identified themes which may provide more in-dept exploration of the quantitative results. The first study evaluated experiences of pre-clinical medical and psychology students shortly after completion of an abridged mindfulness training and reported two main themes “understanding mindfulness” and “engaging in mindfulness,” both influencing each other (Solhaug et al., 2016). Another study in undergraduate medical students reported that a 7-week MBSR training was associated with high levels of satisfaction when delivered on an optional basis (Aherne et al., 2016). A recent qualitative review (Crowther et al., 2020), which included 16 studies evaluating various mindfulness interventions in relatively small cohorts of mainly female undergraduate health and social care students, reported beneficial effects on stress reduction, self-compassion, peer cohesion and support, ability to attend patients by staying present, enhanced listening, insights into health culture, and lifestyle adjustments. However, several issues remain to be considered, including the most suitable moment of this training in the curriculum, short and long-term impact on personal and professional life, and contributing factors of adherence and maintenance of mindfulness practice. Therefore, the aim of the current qualitative study is to explore the nature of mindfulness practice in clinical clerkship students, its possible effects and facilitators and barriers 2 years after their participation in MBSR training.

## Materials and Methods

### Study Design and Setting

A qualitative interview study was performed with clinical clerkship students from the Radboudumc in Nijmegen, Netherlands, who previously participated in a RCT examining the effect of MBSR training on wellbeing (van Dijk et al., 2017). The present study evaluated the long-term effect of MBSR training by interviewing students who ended this training at least 18 months ago about their experiences after the training. Semi-structured interviews were conducted to let the participants talk freely with structured guidance from the interviewer using an interview guide (see Supplementary Material). Individual interviews created the opportunity for students to openly speak their minds without the risk of mutual influence or mainly politically correct answers.

The medical curriculum of the Radboudumc consists of 3 years of preclinical bachelor study and 3 years of master study, which involve rotating through a fixed order of hospital placements alternated with short periods of didactic classroom teaching.

The Consolidated criteria for Reporting Qualitative research (COREQ) (Tong et al., 2007) and the Standards for Reporting Qualitative Research (SRQR) (O’Brien et al., 2014) were applied in reporting the results. The study was approved by the ethical committee of the Radboudumc, Nijmegen (CMO no. 2010/388; ABR no. NL33969.091.10).

### Participants

In the present study, all participants allocated to the intervention group (MBSR training) from the previous RCT (van Dijk et al., 2017) were eligible (*n* = 83). Purposive sampling was used to collect in-depth information from a wide selection of clinical clerkship students. The selection was based on gender, clerkship group, MBSR trainer, time since MBSR and evaluation of training by the students expressed in a grade from 0 to 10 (see Table 1). The aim of purposefully selecting students based on these criteria was to obtain a wide variety in answers during the interviews.

**TABLE 1:** Characteristics of interviewed clerkship students and non-participants from Radboudumc, Nijmegen, Netherlands (2013–2014), 2 years after participation in mindfulness-based stress reduction training.

No.	Gender	Evaluation of training by students (grade 0–10)	Age at time of interview (years)	Time between training and interview (months)
***Participants***				
1	Female	8	27	23
2	Male	8	27	23
3	Female	7	25	31
4	Female	9	29	29
5	Female	6	26	22
6	Male	8	26	31
7	Female	8	31	26
8	Female	7	27	24
9	Female	8	24	25
10	Female	7	24	28
11	Female	7	27	24
12	Male	7	24	22
13	Female	7	23	18
14	Male	9	26	35
15	Female	6	45	35
16	Female	7	25	23
*Mean (SD)*			*27.3 (5.1)*	*26.2 (4.9)*
*Median (range)*		*7 (6–9)*		
***Non-participants***				
1	Female	7	*(No response to phone and email)*	
2	Female	7	*(Not possible to plan appointment)*	

The intervention in the RCT consisted of a MBSR training with eight weekly 2 h sessions (from 4.30 p.m. to 6.30 p.m.). The trainings took place between February 2011 and September 2012 and were taught by a psychiatrist (AS) and a physician, who both met the standards of good practice guidelines for teaching mindfulness-based courses (Crane et al., 2012). The program consisted of formal mindfulness exercises (bodyscan, meditation, yoga), informal exercises during daily life activities, psycho-education about different topics, group dialogue and daily home practice.

### Data Collection

The in-depth face-to-face interviews took place between October 2013 and March 2014. Students were approached by telephone and were informed about the study. Students interested to participate were sent an information letter and after a couple of days an interview was scheduled by telephone.

In general, the interviews were held at the department of Primary and Community Care of the Radboudumc, which was an unfamiliar, neutral area for the students. Due to practical reasons, one interview was conducted by an online video call due to removal and one at the student’s home due to illness.

Prior to the interview, confidentiality was assured and the process of the interview was explained. An interview guide was used containing the main questions related to the research questions. The guide included questions about the long-term effects of MBSR training on personal life, current benefits in professional life, evaluation of the training and barriers and facilitators to maintain mindfulness practice. The interviews were performed by two researchers, took 21–44 min, and were audiotaped after consent. An anonymized short summary of each interview was e-mailed to the student for approval. The coordinating researcher (IvD) kept a log containing notes about the process and content of data collection and analysis. The interview guide underwent minor adaptions based on discussions after each interview as part of the iterative process to maximize the exploration of possible missing topics. The final (fifth) version is provided in Supplementary Material. Data saturation was reached after 16 interviews, meaning that the ability to obtain additional new information was attained.

#### Interviewers

Two female interviewers, not previously known to the students, conducted the individual interviews. The coordinating researcher (IvD) was a 34-years old psychiatrist in training who participated in a 2-year post academic traineeship to become MBSR teacher. The second interviewer was a 40-year old coordinator of healthcare programs in primary care, and a psychologist in training. This interviewer completed an 8-week MBSR and was therefore familiar with mindfulness terminology.

### Analyses

All interviews were fully transcribed. The anonymized transcripts were independently analyzed by two researchers who also conducted the interviews. The analyses were conducted with the aid of qualitative analysis tool of Atlas.ti GmbH Version 7 (Berlin, Germany). A process of constant comparison was used, originating from the grounded theory (Glaser and Strauss, 1967). At first, the researchers independently read and coded the transcribed text of each interview, and stayed semantically close to the participants’ wording (open coding). In a second step, the process of grouping codes into overarching categories was started after coding the first four interviews (axial coding). During this inductive coding process, the researchers discussed the findings until mutual agreement was achieved. Thirdly, connections between categories were examined to form overarching themes (selective coding). The constant iteration of these steps led to a deep understanding of the effects of MBSR training, and the barriers and facilitators in starting and maintaining mindfulness practice in clinical clerkship students. Halfway the process of coding and categorizing, the interim results were discussed at a research meeting for mindfulness researchers of the Radboudumc to get a better understanding of the possible relationships between categories and themes, and to prevent tunnel vision.

## Results

Three main themes related to the nature and effects of students long-term mindfulness practice came forward; (1) *focused attention and open awareness* during daily activities as core elements of long-term mindfulness practice; (2) *changes in behavior and coping* that resulted from practice, such as taking a pause, reflecting and recognizing automatic behavioral patterns; (3) *integration in personal and professional life.*

In total, 18 clinical clerkship students were approached to be interviewed. Two students did not participate: one showed interest initially, but did not respond to follow-up contact while the second agreed to participate but no meeting could be arranged within the requested time period. Table 1 summarizes characteristics of participating and non-participating students.

**FIGURE 1:**
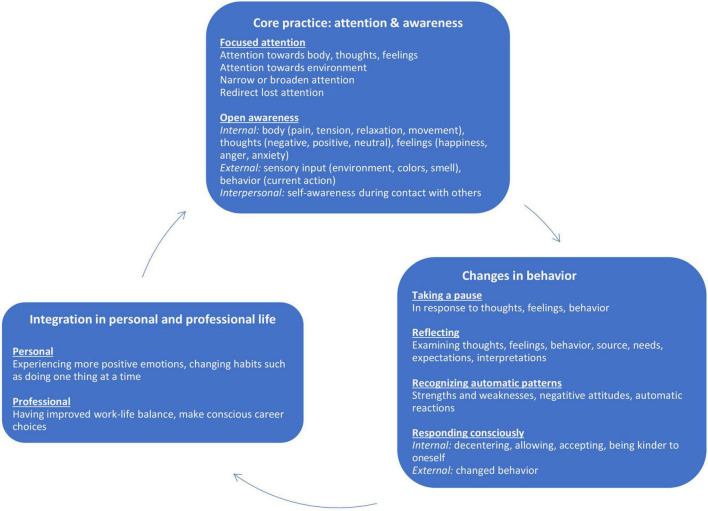
Factors associated with incorporation of mindfulness practice.

The 16 interviews resulted in a total of 155 codes in 42 categories combined in eight subthemes in three main themes (see Figure 1). Some interviewees reported that they had discontinued practice and hence did not notice any changes in lifestyle as a consequence of MBSR training. Usually when an interviewee felt no need, he/she already used other ways of increasing wellbeing such as sports or prayer:

*“It is more that I don’t feel the need for it, as it seems. I’m a Muslim and then you already pray five times a day and have a moment of contemplation and rest, that is already built in for you.”* [Interviewee 14]

Figure 1 visualizes the three themes, subthemes and their relationship. All interviewees elaborated on one or more of these themes. A number of interviewees stated in the beginning of the interview that they did not engage in mindfulness practice anymore because they stopped using formal meditation exercises. However, when they were asked about informal practice they realized that they *had* integrated aspects of mindfulness practice into their life.

### Focused Attention and Open Awareness

The core of students’ long-term mindfulness practice, described by almost all students, existed of consciously paying attention to sensory perceptions during daily activities or using a short mindfulness exercise (e.g., 3 min breathing space). In all cases this focus of attention was directly linked to an enhanced awareness during the activity, for example, when brushing their teeth or taking a walk:

*“When I’m taking a walk in the forest I stand still, take a deep breath and just notice the forest without being occupied with other things.”* [Interviewee 8]

### Changes in Behavior and Coping

Being more aware of a situation supported students in dealing with it differently by taking a pause, reflecting on the situation or by recognizing automatic behavioral patterns:

*“I really thought well yes, I’m always trying to do everything at once. So that was an insightful moment. When I brushed my teeth then I would do three other things while brushing my teeth instead of brushing them consciously.”* [Interviewee 13]

Taking a pause created space for a conscious *internal* response, for example, by allowing emotions instead of repressing them or by decentering from negative thoughts:

“*Well, if I have those crazy thoughts like ‘life isn’t worth living anymore’ then I can think ‘it is just a thought’ and it feels as if I can look at it from a distance instead of diving further into it.”* [Interviewee 4]

Occasionally, students mentioned that they could easier accept a difficult situation or feeling:

*“When experiencing tension or stress, before I wanted it to go away, I didn’t want to feel it. The training taught me another way to deal with it: ‘ok, well, that is the way it is’.”* [Interviewee 1]

A conscious response could also be *external* existing of different behavior, for example, when a computer doesn’t work properly:

“*I just sit back and wait, often you can see on screen that it is ‘thinking’ or that it restarts. Then I just wait until maybe it solves itself. Normally I would have pushed all buttons and would have called someone for help, but now I first just wait.”* [Interviewee 11]

## Integration in Personal and Professional Life

### Personal

Sometimes paying more attention and being more aware of daily activities, *directly* resulted in feeling better and enjoying life more:

*“That you cycle home and just take the time to enjoy the nice ride. That the birds are singing and the sun is shining, those kind of things. That the trees are beautiful, well, those are things that I didn’t really pay attention to in the past.”* [Interviewee 11]

In other situations, quality of life *indirectly* increased as a result of their changed ways of coping, for example, by letting go of dysfunctional patterns:

*“I used to be preoccupied with other people’s opinions of me, so badly that I couldn’t even listen to what they would say. That has become less and less.”* [Interviewee 4]

### Professional

A number of students reported an improved work-life balance as a result of setting more boundaries, expressing their opinion toward superiors and learning not to take work problems home:

*“I became more aware that I have to separate work from private life. Go home without taking work problems home. For example, if I’ve seen a complex patient I first discuss it with a colleague and then put it to rest without taking it home.”* [Interviewee 11]

Some students even explained how the increased awareness of work-life balance supported them in making a conscious career choice:

*“Because of that, I started thinking about if I wanted to work in the hospital or outside the hospital. I noticed that there is a big difference in how people treat each other in the hospital, which I experience as more stressful.”* [Interviewee 9]

## Barriers and Facilitators in Starting and Maintaining Mindfulness Practice

### Preconditions for Practice: Understanding and Intention

#### Understanding

The understanding of what mindfulness is and how it could be integrated into students’ lives influenced their decision whether or not to continue practice after the training. An unrealistic or incorrect understanding discouraged students from practicing, for example, one student defined mindfulness as a way to distract yourself from distress that was not perceived as. useful. For another student gaining a better understanding of mindfulness was the only long-term result reported:

“…*at least I know what it is now and I’m actually happy with that.”* [Interviewee 10]

#### Intention

Next to a realistic understanding, also the intention to practice appeared a precondition, which was related to students’ considering whether now would be “the right time” for practice. A lack of intention resulted in long-term post-poning:

*“Well, what I do think is that in a few years it‘ll possibly do something and then I will at least have the tools and will be able to use them. But I think that now I’m just not ready yet. That’s it.”* [Interviewee 7]

### Factors in Maintaining Mindfulness Practice

#### Practical

Practical aspects such as lack of time and not having a space to practice were often mentioned as barriers for practice. Although students were satisfied that the training took place in the beginning of their clerkships, their busy schedule and lack of autonomy caused them to practice at best around 15 min a day. As a result, some students felt insufficiently equipped to maintain their practice.

#### Personal

As mentioned before, students’ understanding of mindfulness and their intention were the most important factors in whether or not they maintained their practice. Practicing only the formal and informal exercises that they liked most and experiencing positive effects of exercises were additional facilitators while a lack of self-discipline and feeling no need to practice were additional barriers.

#### Professional

Specifically during clerkships, a lack of autonomy, supervisors’ negative opinion about mindfulness and high work pressure were mentioned as reasons for discontinuing mindfulness practice. Being able to select short exercises was a facilitator.

## Discussion

This qualitative study evaluated the nature and effects of mindfulness practice of clinical clerkship students 2 years after participation in an 8-week MBSR training. Three overarching themes as well as several possible facilitators and barriers for starting and maintaining mindfulness practice emerged, giving more insight in which factors contribute to the possible sustainable effects of mindfulness practice in the medical curriculum. The nature of students’ long-term mindfulness practice varied from no practice at all to maintaining regular, mostly informal, practice. Core practice existed of focusing attention on bodily sensations or the environment during daily activities followed by enhanced awareness. In some interviewees this resulted in changed ways of coping like taking a pause, reflecting on the situation or recognizing automatic behavioral patterns, making space for a conscious response. Interviewees described both conscious *internal* responses such as taking distance (decentering) from negative thoughts, as well as *external* responses such as changing behavior toward a clerkship supervisor or patient. Overall, students’ mindfulness practice and their changed ways of behavior or coping enhanced their enjoyment of daily activities, improved their work-life balance and sometimes influenced career choices by opting for other work setting or specialization.

The qualitative findings of the present study on the effect 2 years after a MBSR training in clinical clerkship students are in line with the scarce literature about long-term effects of mindfulness. A RCT in medical students demonstrated the viability of mindfulness training in promoting wellbeing and adaptive coping measured 6 years after the training (de Vibe et al., 2018). A mixed-method study in medical graduates, of whom over half of them indicated to still practice mindfulness up to 10 years after, showed similar results with lower self-reported stress, and better patient and personal connections (Staffaroni et al., 2017).

### Importance of the Understanding of Mindfulness

Having a realistic understanding of mindfulness appeared to be a precondition for maintaining long-term mindfulness practice. This confirms previous findings in first-year medical and psychology students shortly after MBSR; students with an instrumental approach of mindfulness experienced less benefits than those with a more comprehensive understanding (Solhaug et al., 2016).

Although in the current study not all students did develop a long-standing mindfulness practice, having participated in a training could in itself be valuable. Medical students with mindfulness experience have a greater knowledge of and are significantly more likely to administer or recommend it to others than students without exposure to mindfulness (Shapiro et al., 2006).

### Attentional vs. Attitudinal Changes

In current literature, intention (purposefulness), attention (observing internal and external experiences) and attitude (the way one pays attention) are distinguished as three core aspects of mindfulness which are interwoven and occur simultaneously (Shapiro et al., 2006). In the present study, intentional and attentional aspects were clearly present, but changes in the *way* students payed attention (e.g., non-judging, compassionate) were rarely mentioned similar to findings of a study in first-year medical and psychology students (Solhaug et al., 2016). In contrast, in a qualitative study among Dutch medical residents 6 months after MBSR, “acceptance and non-judgment” was one of five central themes (Verweij et al., 2018). Physicians and other healthcare professionals also reported the cultivation of an increasingly open and self-compassionate attitude toward themselves (Irving et al., 2014).

In this study, the less profound attitudinal changes in students might be related to their younger age and lack of work experience. Students were invited to mindfulness training during the core curriculum without selection on distress level, which might have caused them to practice less. Compared to the residents and healthcare professionals who applied for the training themselves, they also might have had less understanding of mindfulness beforehand. Finally, many students only used the exercises they liked most, avoiding impatience and frustration, which might lead to less opportunities to cultivate attitudinal changes. Despite this, it is encouraging that although formal practice after 2 years is rare, students still reported many relevant benefits.

### Strengths and Limitations

Strong aspects of the present study include the use of an unselected population of clerkship students of the original RCT (van Dijk et al., 2017) implying good external validity, and the use of the purposive sampling method for this qualitative study. The MBSR training was taught by qualified medical doctors which may have increased acceptability for the medical students and the standard protocol for MBSR was used. Furthermore, the current findings add to the existing literature, because of the focus on MBSR training in medical clerkship students instead of undergraduates and evaluation of long-term effects. None of the qualitative studies in the review of Crowther et al. evaluated effects of mindfulness in medical clerkships students (Crowther et al., 2020).

A possible limitation of the study was the difficulties some students mentioned in differentiating which changes of behavior could be attributed to the MBSR training and which to their regular personal and professional development.

### Implications for Practice

Considering the high levels of distress, depression and burnout in medical students and physicians (in training), which may adversely affect patient safety, the medical curriculum should provide tools for students to reduce stress and promote positive (mental) health. MBSR could be of particular benefit as highlighted in the present study giving students the opportunity to integrated mindfulness in daily life with possible positive effects on professional functioning.

A main barrier in starting and maintaining mindfulness practice seems the demanding clerkship schedule giving rise to the question what the right timing and format would be for offering medical students a mindfulness-based intervention. MBSR training shortly before the start of clerkships with follow-up meetings every month or two could be an alternative format. The follow-up meeting might support the long-term integration of mindfulness practice in their personal and professional life. Based on our results it seems valuable to actively explore students’ understanding of mindfulness during the training to avoid unrealistic ideas or expectations. Examining attitudes of supervisors and other role-models could be important to prevent students from adapting to their environment and discontinuing mindfulness practice. Although little research has been done on teachers’ perspective and influence, previous research revealed that students identify those who are not competent and specialized sufficiently (Crowther et al., 2020).

### Recommendations for Further Research

A qualitative approach evaluating MBSR complements the mostly quantitative data available, therefore mixed method approaches should be considered in further research.

Although the themes found in the present study are in accordance with previous literature, a more in-depth insight in the development of compassion next to self-awareness through MBSR training may help to facilitate mindfulness practice in daily and professional life. It would be interesting to investigate the effects of compassion training on empathy and self-compassion, either as an additional explicit component in mindfulness-based interventions for medical students or as a stand-alone training following MBSR. Furthermore, in light of the mainly female cohorts investigated before, it would be worthwhile to explore how to make mindfulness more acceptable to students with a more diverse background both in terms of gender and other possible aspects such as cultural, socio-economic background and sexual identity. Customizing MBSR to the specific needs of the student population, like in the Mindfulness-Based Coping With University Life (Lynch et al., 2011), may increase mindfulness practice and may optimize its benefits.

## Conclusion

Despite the busy clerkship schedule, the limited practice of students and the relatively short training period of 8 weeks, many interviewees still engaged in some form of mindfulness practice 2 years after the training. Mindfulness practice strengthened students’ focused attention and self-awareness, enabling changes in behavior and/or coping which could lead to improvements in personal and professional life. In light of the high clerkship demands, MBSR could be a valuable addition to the medical curriculum, supporting students in developing necessary competencies to become well-balanced professionals. Offering a training shortly before clerkships with regular booster sessions over a longer follow-up might better support students in maintaining a long-term practice.

## Data Availability Statement

The raw data supporting the conclusions of this article will be made available by the authors, without undue reservation.

## Ethics Statement

This study involving human participants was reviewed and approved by the medical ethical research committee, Arnhem-Nijmegen (Protocol registration nr. 2010/388 and ABR nr: NL33969.091.10). The participants provided their written informed consent to participate in this study.

## Author Contributions

ID, AS, PL, and CW contributed to conception and design of the study. ID, MvB, and MAJ organized the database. ID performed the statistical analysis under supervision of AS, MvB, and MAJ and wrote the first draft of the manuscript. MvB and MAJ wrote sections of the manuscript. All authors contributed to manuscript revision, read, and approved the submitted version.

## Conflict of Interest

The authors declare that the research was conducted in the absence of any commercial or financial relationships that could be construed as a potential conflict of interest.

## Publisher’s Note

All claims expressed in this article are solely those of the authors and do not necessarily represent those of their affiliated organizations, or those of the publisher, the editors and the reviewers. Any product that may be evaluated in this article, or claim that may be made by its manufacturer, is not guaranteed or endorsed by the publisher.
