# Supplementary material for: Experiences of Clinical Clerkship Students With Mindfulness-Based Stress Reduction: A Qualitative Study on Long-Term Effects
Source: Front Psychol. 2022 Mar 31;13:785090. doi: 10.3389/fpsyg.2022.785090 (PMC9009088; doi:10.3389/fpsyg.2022.785090)
Supplement: Supplementary file 1 [file Data_Sheet_1.pdf]

Appendix 1

Interview guide version 5

<u>Introduction:</u>

Introduction, coffee/tea

Welcome, thank for participation

Interviews are part of overarching Interns InSight Study, on the wellbeing of clinical clerkship students

Ask permission for audio-taping, explain procedure of anonymizing data, state independence of interviewer and expected duration of interview

<u>Topic 1: Effect of MBSR on personal life</u>

1. Which expectations did you have about the training beforehand?
2. Which intention did you have for participation in the training? What did you want to learn?
3. How did you experience the training?
4. How did the home practice go? What were barriers? What was helpful?
5. Which exercises do you remember?
6. Which effect did this exercise have?
7. In general, did you pick up something useful from the training or not? If so, what? When? How? With what consequence? In which situation? With which effect?
8. How did you learn that? Which part of the training was (un)helpful?
9. How is your mindfulness practice now compared to shortly after the training? Are there differences between now and then? How did it go over time?
10. How did you continue after the training?
11. Do you still use anything from the training? Informal exercises? Which? Where? When? How? With which consequence?
12. Are there aspects of the training that you have integrated in daily life? Which, where, when, how? With which consequence?
13. What were barriers and facilitators in this process?

<u>Topic 2: Effect of training on professional life</u>

1. Are there any aspects of the training that you use in professional life? (if present, ask for the before mentioned experiences in personal life)
2. Can you give an example? Can you tell something more about that? (what, where, when, how?)
3. *If it is unclear for a student what kind of examples you mean*: give example of application in stressful situation

4. *If a student does not engage in mindfulness practice during work but does give examples of mindfulness practice in personal life:* Do you know why there is a difference between mindfulness practice in your personal and professional life? What could be the cause of that? Could you give an example?
   Ask students about their mindfulness practice related to specific topics that were addresses during the training:
5. Stressful situations?
6. Communication with patients or colleagues?
7. Accepting and allowing emotions?
8. Work-life balance?

Topic 3: Evaluation of the training

1. Looking back, what do you think of the structure of the training?
2. The duration?
3. The shape?
4. The timing (at that time during clerkships)?
5. The teachers?
6. After the training, did you have an idea about how to integrate mindfulness practice in daily life or professional life? If not, how could the training be improved to support that?
7. Home practice: if practiced little, what would have supported home practice?
8. Does a mindfulness training add something to the curriculum or not?
9. Can you give an example? Tell something more about that?
10. Any suggestions for adaptations to the training?

If the interviewee did not benefit from the training:

1. Did you have any expectations about the effects of the training? What was the aim for you? In what way did the training not meet that expectations?
2. Explain difference between formal and informal practice and ask about informal practice
3. Is there a way in which you could have benefitted more from the training? How? Adaptation of training?

Closure:

Any questions? remarks?

Thank for participation. Check e-mail address for sending summary (member check)
